# The alterations in event-related potential responses to pain empathy in breast cancer survivors treated with chemotherapy

**DOI:** 10.3389/fpsyg.2022.942036

**Published:** 2022-09-23

**Authors:** Wen Li, Yue Lv, Xu Duan, Guo Cheng, Senbang Yao, Sheng Yu, Lingxue Tang, Huaidong Cheng

**Affiliations:** ^1^Department of Oncology, The Second Affiliated Hospital of Anhui Medical University, Hefei, China; ^2^Department of Finance, University of Connecticut, Storrs, CT, United States

**Keywords:** breast cancer, pain empathy, chemotherapy, event-related potentials (ERP), cognitive

## Abstract

**Background:**

Previous findings indicated that breast cancer patients often have dysfunction in empathy and other cognitive functions during or after chemotherapy. However, the manifestations and possible neuro-electrophysiological mechanisms of pain empathy impairment in breast cancer patients after chemotherapy were still unknown.

**Objective:**

The current study aimed to investigate the potential correlations between pain empathy impairment and event-related potentials (ERP) in breast cancer patients undergoing chemotherapy.

**Methods:**

Twenty-two breast cancer patients were evaluated on a neuropsychological test and pain empathy paradigm before and after chemotherapy, containing the Chinese version of the Interpersonal Reactivity Index (IRI-C), while recording ERP data.

**Results:**

The empathic concern scores were lower and personal distress scores were higher on IRI-C task compared with those before chemotherapy (*t* = 3.039, *p* < 0.01; *t* = −2.324, *p* < 0.05, respectively). Meanwhile, the accuracy rates were lower than those before chemotherapy for both pain and laterality tasks on the pain empathy paradigm (*F* = 5.099, *P* = 0.035). However, the response time was no significant differences before and after chemotherapy (*F* = 0.543, *P* = 0.469). Further, the amplitude of the N1 component was significantly increased (*F* = 38.091, *P* < 0.001), and the amplitude of the P2 component was significantly decreased (*F* = 15.046, *P* = 0.001) in the subsequent ERP study. A linear mixed effect model was used to analyze the correlation, the average amplitude of N1 and P2 were positively correlated with the accuracy rates in laterality tasks (*r* = 1.765, *r* = 1.125, respectively, *P* < 0.05).

**Conclusion:**

The results indicated that pain empathy impairment was performed in chemotherapeutic breast cancer patients, which was possibly correlated to the changes of N1 and P2 components in ERP. These findings provide neuro-electrophysiological information about chemo-brain in breast cancer patients.

## Introduction

Breast cancer was the most frequent malignancy in women, occupying 30% of all new cancer cases diagnosed in women from the published data of the American Cancer Society (Siegel et al., 2021). The incidence of breast cancer in women was estimated to be increasing slowly by 0.5% per year, which contributed to 287,850 new cases of invasive breast cancer diagnoses and 43,250 deaths cases from breast cancer recorded among United States women in 2021 (Siegel et al., 2022). Like the United States, China has a huge number of breast cancer patients; the highest incidence of malignant tumor in women was still breast cancer, with about 304,000 new cases every year, which means the treatment and quality of life of breast cancer survivors are worth our extensive attention (Cao et al., 2020). Breast cancer is a highly heterogeneous malignant tumor, and although treatment options have certain differences, chemotherapy was still one of the main treatment methods for breast cancer, which could improve the efficacy and prognosis of patients (Krajnak et al., 2022). However, while chemotherapy could bring good news to patients, the adverse reactions should be of concern – many chemotherapy drugs, especially anthracycline, can directly or indirectly pass through the blood-brain barrier and produce toxic effects on the central nervous system, leading to neurocognitive impairments for breast cancer patients (Wefel et al., 2015). Anthracycline could be accumulated in the nucleus of neurons, which results in DNA double-strand breaks, causing the death of neurons and seriously affecting the normal cognitive activities of the brain (Manchon et al., 2016). This performance was called chemotherapy-related cognitive impairment (CRCI) or “chemo brain,” which was used to refer to the increasing forgetfulness, changes in emotion, trouble with concentrating, and impaired multitasking ability (Shaw et al., 2021). Breast cancer patients are not only worried about the progression and recurrence of the illness itself but also expressed serious anxiety and fear about the cognitive impairment and future quality of life after receiving chemotherapy, which results in yet further emotional distress (Simmons, 2009).

Cancer patients tended to have more emotional problems in terms of pain, such as fear and sadness in comparison with healthy individuals (Bultz et al., 2015). Empathy is not only the temporary state of emotional cognition but also a relatively constant personality trait, which plays extremely important positive effects on individuals and in society (Rae et al., 2020). Pain empathy is a common component of emotion, which focused on understanding and experiencing others’ painful feelings and responding to them emotionally and behaviorally (Mu et al., 2021). When doctors see patients suffering from pain, they experience compassion, so pain empathy has often been used to measure the professional ethics of medical staff (Roche and Harmon, 2017). Of course, pain empathy is not unique to mankind, which was also common in rodents, and further investigation found that pain empathy was related to the anterior cingulate cortex (Smith et al., 2021). Compared to animal empathy, our own pain empathy is influenced by all kinds of psychosocial factors and in turn became awfully complex (Mouras and Lelard, 2021).

Event-related potential is a highly sensitive and non-invasive technique, which is commonly used in the area of psychology and cognitive neuroscience, with an accuracy of up to milliseconds, and can permit the evaluation of pain empathy (Kappenman et al., 2021). Breast cancer patients revealed attention and information processing disorders when receiving chemotherapy, and significant changes in ERP components had been observed (Kam et al., 2016). Our team proved that breast cancer patients presented poor emotional regulation ability after chemotherapy, which might be related to the changes of N1, P2, and N2 components for ERP (Gan et al., 2019). Some research indicated that pain empathy was correlated to the different components of ERP, in which early selective attention was related to N1 and the recognition and processing of stimulus were relevant to P2 (Yang et al., 2017). The early N2 differentiation was observed in the frontal lobe between painful and painless stimuli under moderate and low competitive intensity conditions (Luo et al., 2018). Other findings indicated that participants extracted more positive frontal-central N1 and N2 amplitude when hearing others’ voices in pain compared with perceiving others’ neutral voices (Liu et al., 2019). Therefore, the ERP components related to pain empathy were different with the change of research objects and conditions, which indicates that pain empathy was susceptible to multiple factors.

Breast cancer patients would feel inferior and depressed due to the incomplete body after radical mastectomy, meanwhile, chemotherapy could also reduce their concern for surrounding things, resulting in emotional regulation and empathy impairment, which would even persist for several years (Conley et al., 2016). However, most empathy research focuses on providing more humane medical services to relieve anxiety, depression, and other psychological symptoms, without paying attention to the essence of empathy impairments in cancer patients (Laverdiere et al., 2019). Although pain empathy impairment has a significantly negative impact on all aspects of daily and social life in breast cancer patients after chemotherapy, what are the specific variations and the related neuro-electrophysiological mechanisms for pain empathy impairment? At present, the research content of these aspects is almost a blankpage.

In this study, 22 breast cancer patients who accept chemotherapy were enrolled and cognitive test, pain empathy paradigm, and ERP studies were completed before and after chemotherapy, trying to confirm the specific performance on pain empathy, to further clarify the correlations between pain empathy impairment and ERP in breast cancer patients undergoing chemotherapy.

## Materials and methods

### Participants

Twenty-two patients with breast cancer were volunteered from 2017 to 2019 in the Department of Oncology, the Affiliated Second Hospital of Anhui Medical University. The Research Ethics Committee of the Affiliated Second Hospital of Anhui Medical University, China, authorized the study (protocol 20131028). The informed consent were obtained for all subjects. All participants had normal vision and could cooperate well with the inspection. Inclusion criteria: (1) Diagnosed with breast cancer and completing at least six cycles of chemotherapy; (2) Anthracycline-based chemotherapy regimens; (3) No language or other communication barriers; (4) normal daily activities could be acquired, KPS scores ≥80. Exclusion criteria: (1) history of psychotropic medications; (2) dementia, anxiety, and/or other psychiatric illnesses; (3) a history of radiotherapy and endocrine therapy; (4) brain metastases; (5) Abuse of alcohol or illicit drugs.

### Cognitive tasks

Participants took part in the following tasks before and after chemotherapy. A mini mental state examination (MMSE) which included orientation, memory, attention, computation, recall, and language served as a screening tool for overall cognition, for which the total possible score was 30 points (Mintun et al., 2021). Digit spans tests (DST) were the usual method to evaluate short-term verbal memory ability, including two portions, DSF, and DSB (Baddeley, 2003). The VFT required the subjects to name as many animals or vegetables as possible within 30 s without repeating them, which could be used to evaluate the language production speed, language memory, and executive function (Andryszak et al., 2017). The Chinese version of the Interpersonal Reactivity Index (IRI-C) was applied to detect empathy ability in participants, including four parts, which respectively involve perspective taking (PT), empathic concern (EC), fantasy, and personal distress (PD). A Likert five-point scoring method was adopted. The higher the score was, the better the empathy ability obtained (Yao et al., 2021).

### Pain empathy paradigm

Add up to 120 pictures were applied to the pain empathy paradigm, depicting human hands and feet under painful or neutral stimulation, but without facial expressions. All painful stimulation events could happen at any time in daily life, such as catching the left (or right) hand in closing doors and windows, cutting the left (or right) fingers on a kitchen knife, and other daily mishaps.

The pain empathy paradigm consisted of pain and laterality tasks. The participants were required to determine whether the people in the photo felt pain and hit the corresponding computer key (F for pain, J for no pain) in the pain task. Additionally, participants were required to judge the left and right hands or feet being shown in the painful stimulation picture and to hit the corresponding computer key (F indicating the left, J indicating the right) in the laterality task. Each task included 60 pained and 60 neutral photos. Each trial required participants to make judgments as quickly and accurately as possible over a random period of time between 1500 and 1700 ms. The participants were trained by practicing the tasks for 20 pictures before the experiment proper began under the direction of a laboratory technician. Meanwhile, the ERP data was recorded on the pain empathy paradigm task (Gu et al., 2010).

### Event-related potentials recording and analysis

The ERP of the subjects was collected on a Neuroscan recording system (NeuroScan, Sterling, VA, United States) through the 64 electrode cap in this experiment. The reference electrode was positioned at the left mastoid of the subjects, and the grounding lead was attached to the central point of the forehead, recording the vertical and horizontal electrooculograms. The impedances of the skin and electrode needed to be adjusted to less than 10 kΩ, and the sampling rate was 1000 Hz, filtering broadband was 0.05–100 Hz. The digital Filtering channel was 32 Hz/24 (dB/Octave), and the length of analysis time was the appearance of stimulus from the first 200 ms to the later 1000 ms, and removing artifact proceeded on amplitudes exceeding ±100 μV. The mean value of electrode signals at the bilateral mastoid process was regarded as a reference, all electrooculograms were rectified by correlation regression analysis. Referring to previous research and the grand-average potential of each task condition, N1 (50–150 ms) and P2 (150–200 ms) were analyzed for ERP data in this study, and two frontal (fz, fzc) and central electrodes (cz) were chosen for statistical evaluation of the ERPs.

### Statistical analysis

The SPSS software (version 22.0^1^; Chicago, IL, United States) was administered for statistical analysis. Student’s *t*-tests were performed for baseline information. Paired-sample *t*-tests were performed for cognitive testing pre- and post-chemotherapy. The reaction time and accuracy rate for pain and laterality tasks were committed to repeated-measures ANOVAs with time, task, and stimuli as within-subject factors. Similarly, the repeated-measures ANOVAs was also performed for the N1 and P2 component of ERP, and four within-subject factors refer to time point, task, and stimuli as well as electrodes (fz vs. fcz vs. cz). All the results were corrected according to the Greenhouse-Geisser method. A linear mixed effect model was conducted to analyze the correlation via MLwiN2.30 software. SAS/STAT software (version 9.4) was applied to draw forest plots. Statistical significance was described as *P* < 0.05.

## Results

### Clinical characteristics and cognitive tests

**FIGURE 1:**
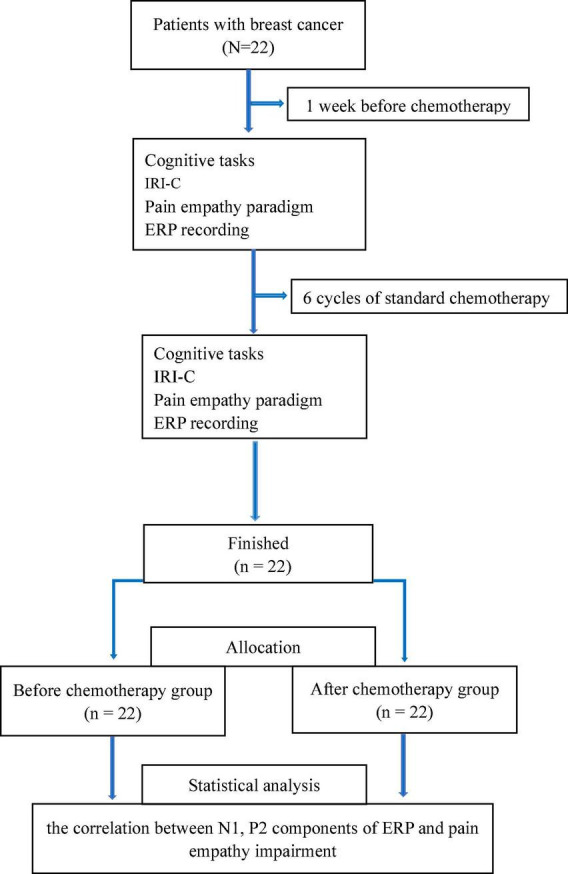
Research flowchart.

Table 1 and Figure 1 contain a sum of 22 patients who were confirmed to meet the criteria; the mean age of the enrolled patients was 50.2 ± 6.0 years, of which 9 patients were over 50 years and 13 patients were under 50 years.

**TABLE 1:** The basic clinical characteristics.

Contents	Numbers/Mean ± SD
Age (year)	50.20 ± 6.00
>50	9 (40.9%)
≤50	13 (59.1%)
Education (year)	7.10 ± 3.00
KPS	90.5 ± 5.80
Chemotherapy regimen	
EC	4 (18.2%)
EC-P/TEC	18 (81.8%)

KPS, Karnofsky Performance Status Scale; EC, cyclophosphamide + epirubicin/doxorubicin; P, paclitaxel; TEC, Taxotere + pirarubicin + cyclophosphamide.

Years of education (7.1 ± 3.0) and KPS (90.5 ± 5.8) are shown for these participants.

Table 2 revealed that the DSB scores became lower from before (4.82 ± 1.56) to after (4.18 ± 1.22) chemotherapy, and had a significant difference (*P* < 0.05). The MMSE (28.68 ± 1.21 vs. 28.50 ± 1.10, *P* > 0.05), VFT (12.27 ± 2.69 vs. 12.13 ± 2.47, *P* > 0.05) and DSF (7.91 ± 0.29 vs. 7.64 ± 0.58, *P* > 0.05) showed no significant difference before and after chemotherapy.

**TABLE 2:** The performance of neuropsychological tests before and after chemotherapy.

	*N*	MMSE	VFT	DSF	DSB
Before	22	28.68 ± 1.21	12.27 ± 2.69	7.91 ± 0.29	4.82 ± 1.56
After	22	28.50 ± 1.10	12.13 ± 2.47	7.64 ± 0.58	4.18 ± 1.22*

*P < 0.05.

MMSE, mini-mental state examination; VFT, verbal fluency test; DSF, digit span forward; DSB, digit span backward.

The IRI-C scores were lower on empathic concern (10.27 ± 2.66, *p* < 0.01) and higher on personal distress (10.14 ± 4.10, *p* < 0.05) as compared to those before chemotherapy (11.68 ± 3.64, 9.05 ± 4.21, respectively), and had a significant difference (Table 3). The scores on fantasy (10.14 ± 3.75 vs. 9.64 ± 2.80, *p* > 0.05) and perspective taking (9.68 ± 3.80 vs. 9.05 ± 3.46, *p* > 0.05) had no significant differences before and after chemotherapy.

**TABLE 3:** Empathy scale results before and after chemotherapy.

		Chinese version of Interpersonal Reactivity Index (IRI-C)			
	***N***	**PT**	**FS**	**EC**	**PD**
Before	22	9.68 ± 3.80	10.14 ± 3.75	11.68 ± 3.64	9.05 ± 4.21
After	22	9.05 ± 3.46	9.64 ± 2.80	10.27 ± 2.66**	10.14 ± 4.10*

*P < 0.05, **P < 0.01.

PT, perspective taking; FS, fantasy; EC, empathic concern; PD, personal distress.

### The results of pain empathy paradigm

As revealed in Table 4, the pain empathy paradigm results were performed through repeated-measure ANOVAs. The accuracy rates were lower than those before chemotherapy for both pain and laterality tasks on the pain empathy paradigm (*F* = 5.099, *P* = 0.035), with a statistical difference. The main effect of the task was significant (*F* = 34.558, *P* < 0.001), and the accuracy of the laterality tasks was higher than that of the pain task (0.91 ± 0.09 vs. 0.88 ± 0.08; 0.87 ± 0.09 vs. 0.80 ± 0.10). The main effect of stimuli was significant (*F* = 37.976, *P* < 0.001), and the accuracy rate of pain stimuli was lower than that of neutral stimuli (0.87 ± 0.09 vs. 0.91 ± 0.09; 0.80 ± 0.10 vs. 0.88 ± 0.08), and there was no significant interaction in time (before vs. after chemotherapy) × stimuli (neutral vs. pain pictures) × task (pain vs. laterality) (*F* = 1.379, *P* = 0.253). However, the response time was no significant difference before and after chemotherapy (*F* = 0.543, *P* = 0.469), the main effect of the task was significant (*F* = 15.540, *P* = 0.001), while there was no significant interaction between time, task, and stimulus (*F* = 1.292, *P* = 0.269).

**TABLE 4:** Statistical analysis of the pain empathy paradigm.

Outcome	Time		Laterality task			Pain task		

			Neutral		Pain	Neutral		Pain
AR (%)	Before		0.96 ± 0.05		0.93 ± 0.05	0.92 ± 0.05		0.81 ± 0.14
	After		0.91 ± 0.09		0.87 ± 0.09	0.88 ± 0.08		0.78 ± 0.10
RT (ms)	Before		644.37 ± 46.95		642.97 ± 45.35	650.35 ± 79.31		664.10 ± 35.24
	After		626.76 ± 42.64		628.68 ± 45.58	669.97 ± 44.73		653.65 ± 67.91
	**Time**	**Task**		**Stimuli**	**Time × task**	**Time × stimuli**	**Task × stimuli**	**Time × task × stimuli**
AR (%)	*F* = 5.099	*F* = 34.558		*F* = 37.976	*F* = 0.400	*F* = 0.543	*F* = 6.863	*F* = 1.379
	*P* = 0.035*	*P* < 0.001**		*P* < 0.001**	*P* = 0.534	*P* = 0.469	*P* = 0.016*	*P* = 0.253
RT (ms)	*F* = 0.543	*F* = 15.540		*F* = 0.056	*F* = 1.603	*F* = 1.549	*F* = 0.000	*F* = 1.292
	*P* = 0.469	*P* = 0.001**		*P* = 0.815	*P* = 0.219	*P* = 0.227	*P* = 0.991	*P* = 0.269

*P < 0.05, **P < 0.01.

Within-subject effects (DF = 1, 21).

AR, accuracy rate; RT, reaction time.

### Event-related potentials

**FIGURE 2:**
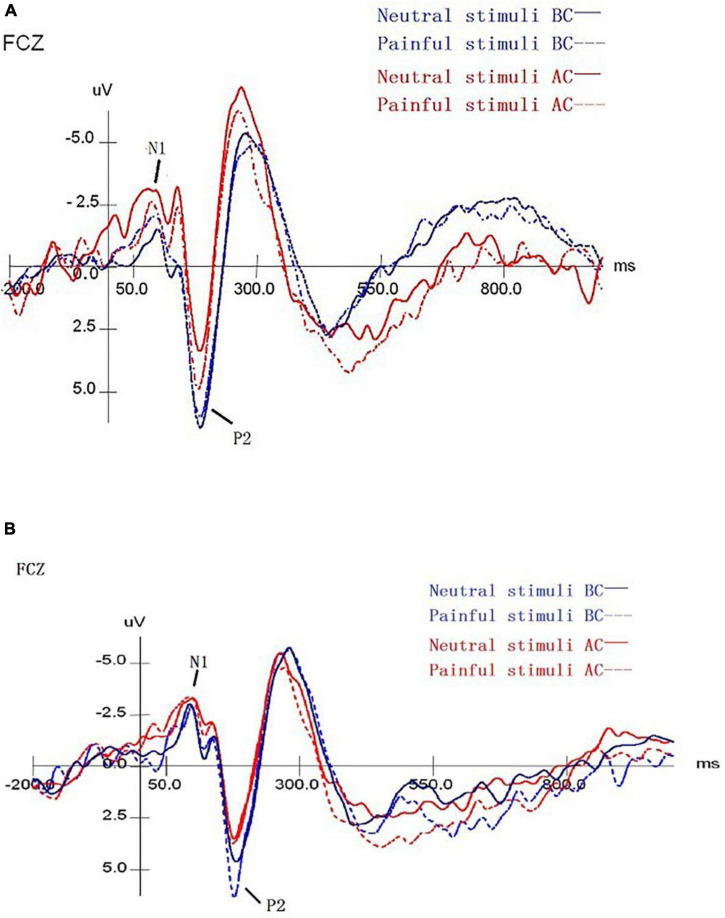
The mean amplitude results of ERP for pain empathy task. **(A)** The mean ERP amplitude of the laterality task before and after chemotherapy. **(B)** The mean ERP amplitude of the painful task before and after chemotherapy. Blue/red lines represent the findings before/after chemotherapy. The solid/dotted lines represent the judgments of neutral/pain stimuli blocks. BC, before chemotherapy; AC, after chemotherapy.

As shown in Figure 2, blue indicates the results before chemotherapy, red indicates the results after chemotherapy, while a solid line represents the judgment of the neutral stimuli picture, and dotted lines represent painful results. Combining Figure 2 and Table 5, we found that the peak amplitude of N1 in patients with pain and laterality tasks was significantly higher in comparison to those before chemotherapy, and had a statistically significant difference (*F* = 38.091, *P* < 0.001). The interactive effect was found in time and stimuli (*F* = 11.056, *P* = 0.004). There were also interaction effects among time × stimulus × task (*F* = 5.169, *P* = 0.035), and the main effects of task, stimuli, and electrode were not significant in the results. As for the P2 component, the peak amplitude after chemotherapy was significantly smaller in comparison to these before chemotherapy, showing a statistically significant difference (*F* = 15.046, *P* = 0.001). The main effect of the stimuli was significant (*F* = 4.889, *P* = 0.039), which means that the pain picture stimulation induced greater amplitude, resulting in a pain effect. There was no interaction between stimulus, task, and electrode.

**TABLE 5:** Statistical analysis for N1, P2 before and after chemotherapy.

Source of variation	N1		P2	
		
	*F*	*P*	*F*	*P*
Time	38.091	0.001**	15.046	0.001**
Task	3.580	0.074	0.581	0.455
Stimuli	0.020	0.889	4.889	0.039
Electrode	5.009	0.037*	4.433	0.049*
Time × task	4.035	0.059	1.938	0.180
Time × stimuli	11.056	0.004**	1.602	0.221
Task × stimuli	0.908	0.353	0.044	0.836
Time × task × stimuli	5.169	0.035*	1.452	0.243
Time × electrode	0.281	0.602	0.026	0.875
Task × electrode	0.544	0.470	0.138	0.715
Time × task × electrode	0.067	0.799	0.128	0.724
Stimuli × electrode	0.001	0.980	0.030	0.864
Time × stimuli × electrode	0.035	0.854	0.367	0.552
Task × stimuli × electrode	0.032	0.859	0.411	0.529
Time × task × stimuli × electrode	0.007	0.935	0.043	0.838

*P < 0.05, **P < 0.01. Bolded values represents a statistically significant difference.

Within-subject effects (DF = 1, 21).

### The correlation analysis between N1, P2, and pain empathy task

**FIGURE 3:**
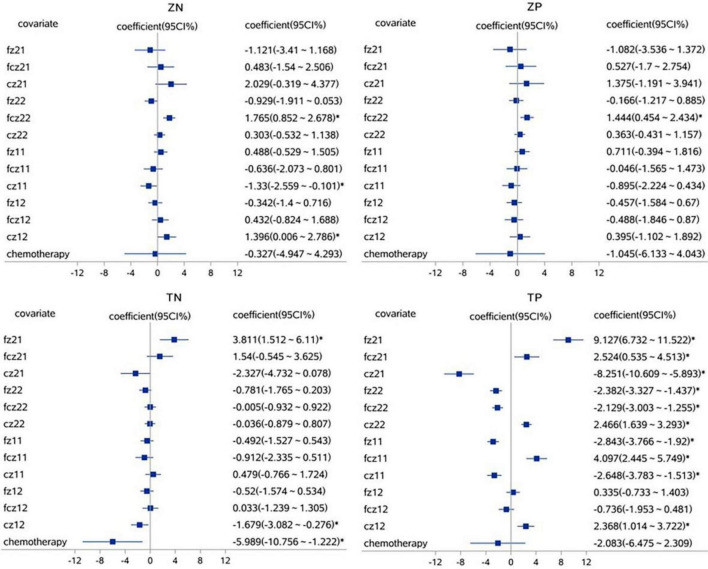
The correlations analysis between ERP N1 component and accuracy rates on pain empathy paradigm. *P < 0.05, linear mixed effect model, when 95% confidence interval (95 CI%) contained no 0 in the forest plots, that is, the minimum value for the interval was positive or the maximum was negative, then *P* < 0.05. fz, fcz, and cz were defined as electrode points for ERP. Z, laterality tasks; T, pain task; N, neutral picture stimulation; P, pain picture stimulation. 21, laterality tasks + pain picture stimulation; 22, laterality tasks + neutral picture stimulation; 11, pain task + pain picture stimulation; 12, pain task + neutral picture stimulation.

**FIGURE 4:**
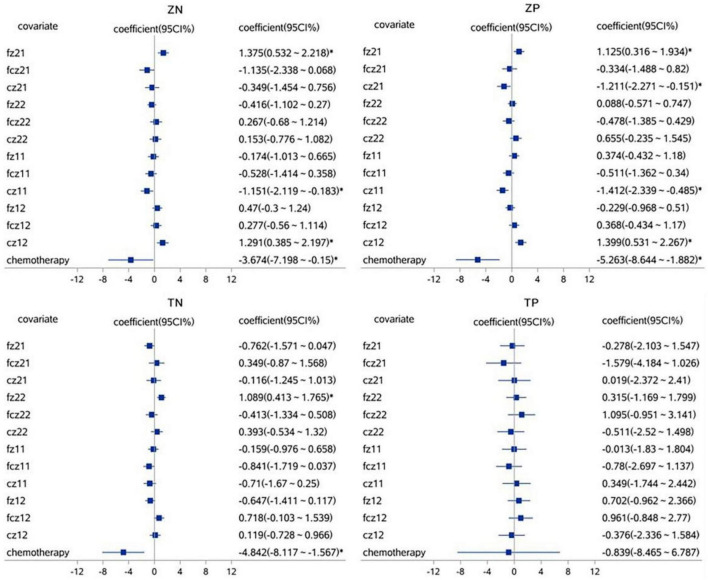
The correlations analysis between ERP P2 component and accuracy rates on pain empathy paradigm. *P < 0.05, linear mixed effect model, when 95% confidence interval (95 CI%) contained no 0 in the forest plots, that is, the minimum value for the interval was positive or the maximum was negative, then *P* < 0.05. fz, fcz, and cz were defined as electrode points for ERP. Z, laterality tasks; T, pain task; N, neutral picture stimulation; P, pain picture stimulation. 21, laterality tasks + pain picture stimulation; 22, laterality tasks + neutral picture stimulation; 11, pain task + pain picture stimulation; 12, pain task + neutral picture stimulation.

A linear mixed effect model was used to analyze the correlation, the average amplitude of N1 at the fcz electrode was positively correlated with the accuracy rates for neutral picture stimulation in laterality tasks (*r* = 1.765, *P* < 0.05). The mean amplitude of N1 at the fcz, fz, and cz electrodes were correlated with the accuracy rates for painful picture stimulation in the pain task (*r* = 4.097, *r* = −2.843, *r* = −2.648, respectively, *P* < 0.05). The mean amplitude of N1 at the cz electrode was correlated with accuracy rates for neutral picture stimulation in the pain task (*r* = −1.679, *P* < 0.05). Similarly, the amplitude of P2 at the fz electrode was positively correlated with the accuracy rates for painful picture stimulation in laterality tasks (*r* = 1.125, *P* < 0.05) (Figures 3, 4 and Tables 6, 7).

**TABLE 6:** The correlations analysis between ERP N1 component and accuracy rates on pain empathy paradigm.

	ZN	ZP	TN	TP
**Fixed parameters**				
fz21	−1.121 (1.168)	−1.082 (1.252)	3.811 (1.173)*	9.127 (1.222)*
fcz21	0.483 (1.032)	0.527 (1.136)	1.540 (1.064)	2.524 (1.015)*
cz21	2.029 (1.198)	1.375 (1.309)	−2.327 (1.227)	−8.251 (1.203)*
fz22	−0.929 (0.501)	−0.166 (0.536)	−0.781 (0.502)	−2.382 (0.482)*
fcz22	1.765 (0.466)*	1.444 (0.505)*	−0.005 (0.473)	−2.129 (0.446)*
cz22	0.303 (0.426)	0.363 (0.405)	−0.036 (0.430)	2.466 (0.422)*
fz11	0.488 (0.519)	0.711 (0.564)	−0.492 (0.528)	−2.843 (0.471)*
fcz11	−0.636 (0.733)	−0.046 (0.775)	−0.912 (0.726)	4.097 (0.843)*
cz11	−1.330 (0.627)*	−0.895 (0.678)	0.479 (0.635)	−2.648 (0.579)*
fz12	−0.342 (0.540)	−0.457 (0.575)	−0.520 (0.538)	0.335 (0.545)
fcz12	0.432 (0.641)	−0.488 (0.693)	0.033 (0.649)	−0.736 (0.621)
cz12	1.396 (0.709)*	0.395 (0.764)	−1.679 (0.716)*	2.368 (0.691)*
Random parameters				
Subjects	5.154 (2.525)*	0.000 (0.000)	0.000 (0.000)	336.071 (107.404)*
Time	25.198 (6.147)*	32.048 (7.166)*	28.127 (6.289)*	15.385 (4.560)*

*P < 0.05, linear mixed effect model, Z-score test was performed for the regression coefficients/(standard error). If Z-score value > 1.96, then *P* < 0.05. fz, fcz, and cz were defined as electrode points for ERP.

Z, laterality tasks; T, pain task; N, neutral picture stimulation; P, pain picture stimulation. 21, laterality tasks + pain picture stimulation; 22, laterality tasks + neutral picture stimulation; 11, pain task + pain picture stimulation; 12, pain task + neutral picture stimulation.

**TABLE 7:** The correlations analysis between ERP P2 component and accuracy rates on pain empathy paradigm.

	ZN	ZP	TN	TP
**Fixed parameters**				
fz21	1.375 (0.430)*	1.125 (0.413)*	−0.762 (0.413)	−0.278 (0.931)
fcz21	−1.135 (0.614)	−0.334 (0.589)	0.349 (0.622)	−1.579 (1.329)
cz21	−0.349 (0.564)	−1.211 (0.541)*	−0.116 (0.576)	0.019 (1.220)
fz22	−0.416 (0.350)	0.088 (0.336)	1.089 (0.345)*	0.315 (0.757)
fcz22	0.267 (0.483)	−0.478 (0.463)	−0.413 (0.470)	1.095 (1.044)
cz22	0.153 (0.474)	0.655 (0.454)	0.393 (0.473)	−0.511 (1.025)
fz11	−0.174 (0.428)	0.374 (0.411)	−0.159 (0.417)	−0.013 (0.927)
fcz11	−0.528 (0.452)	−0.511 (0.434)	−0.841 (0.448)	−0.780 (0.978)
cz11	−1.151 (0.494)*	−1.412 (0.473)*	−0.710 (0.490)	0.349 (1.068)
fz12	0.470 (0.393)	−0.229 (0.377)	−0.647 (0.390)	0.702 (0.849)
fcz12	0.277 (0.427)	0.368 (0.409)	0.718 (0.419)	0.961 (0.923)
cz12	1.291 (0.462)*	1.399 (0.443)*	0.119 (0.432)	−0.376 (1.000)
Random parameters				
Subjects	0.000 (0.000)	0.000 (0.000)	44.869 (14.669)*	0.000 (0.000)
Time	22.514 (5.034)*	20.715 (4.632)*	17.493 (4.479)*	105.404 (233.569)*

*P < 0.05, linear mixed effect model, Z-score test was performed for the regression coefficients/(standard error). If Z-score value > 1.96, then *P* < 0.05. fz, fcz, and cz were defined as electrode points for ERP.

Z, laterality tasks; T, pain task; N, neutral picture stimulation; P, pain picture stimulation. 21, laterality tasks + pain picture stimulation; 22, laterality tasks + neutral picture stimulation; 11, pain task + pain picture stimulation; 12, pain task + neutral picture stimulation.

## Discussion

The present study examined changes in pain empathy in breast cancer patients before and after chemotherapy by using the IRI-C scale and pain empathy paradigm with a concurrent recording of ERP. The results indicated that: (1) Breast cancer patients after chemotherapy achieved significantly lower scores in DSB tests, lower empathic concern, and higher personal distress as compared to the baseline; (2) Breast cancer patients showed a lower accuracy rate after chemotherapy treatment and for painful pictures stimulation as compared to neutral images in both pain and laterality task; (3) The ERP study indicated that the amplitude of N1 component was significantly increased and the amplitude of P2 component was significantly decreased after chemotherapy as compared to before chemotherapy; (4) The positive correlations was confirmed between the average amplitude of N1, P2 and pain empathy through linear mixed effect model analysis.

Empathy involves different components, of which cognitive and affective empathy are relatively independent parts (Zaki and Ochsner, 2012). Among them, affective empathy was the feeling of others’ emotions and the similar emotional resonance in oneself, of which empathic concern and fantasy and personal distress are the components (Choi and Watanuki, 2014). The results of the IRI-C scale in this study suggested that cognitive empathy was intact, but there was an affective empathy disorder, which was manifested as a significant decrease in empathic concern and an increase in personal distress. Our own personal distress response could be triggered when observing other people’s pain (Coll et al., 2017). Affective empathy disorder, on the other hand, could lead to insensitivity to pain. Pain levels were also often underestimated or misassessed in cognitively impaired populations because they could not be effectively expressed (Goubert et al., 2005). This study preliminarily found that the ability of affective empathy was decreased after chemotherapy in breast cancer patients, which performed a theoretical foundation for pain empathy impairment research.

The lower accuracy rate was performed in both pain and laterality tasks on the pain empathy paradigm in this study. Possible reasons were as follows. The modification of brain structure and function appeared in breast cancer patients after chemotherapy, which might cause associated cognitive dysfunction (de Ruiter et al., 2021; Mampay et al., 2021). McDonald et al. (2013) reported that the gray matter density was reduced in some brain regions within 1 month after chemotherapy in breast cancer patients, especially in the prefrontal region. Deprez et al. (2012) found that the gray matter volume of the prefrontal cortex, hippocampus, and cerebellum decreased significantly in breast cancer patients after chemotherapy, and the white matter integrity also declined in the frontal lobe, parietal lobe, and occipital lobe. Gu et al. (2012) found that observing pain in others activated the anterior insula, anterior cingulate, and middle cingulate cortex, which were associated with empathic responses. Imaging evidence indicated that pain empathy was related to the activation of the frontoparietal, temporal, and subcortical regions (Zaki and Ochsner, 2012; Kral et al., 2017; Xiong et al., 2019). Therefore, the prefrontal lobe, inferior frontal gyrus, hippocampus, and other related brain regions of breast cancer patients after chemotherapy might be damaged to varying degrees, while the brain regions related to pain empathy activation were also implicated. Hence, it could be boldly speculated that pain empathy impairment in breast cancer patients after chemotherapy could possibly be related to the structural and functional impairment of the same brain region. In the following studies, Neuroimaging technology will be combined to further clarify the specific brain areas.

Breast cancer patients exhibited a lower accuracy rate in judging images of pain stimuli than neutral stimuli in this study, both before and after chemotherapy. This might be related to the fact that the identification, evaluation, and processing of painful stimuli were more complicated than neutral for individuals. The study found that more areas of the brain were activated in response to painful stimuli than in comparison to neutral stimuli (Luo et al., 2018). The corresponding brain areas were activated by different pain positions, with sensorimotor areas mobilized through limb pain images, while facial pain pictures could more strongly stimulate the midline frontal and parietal cortices and amygdala (Vachon-Presseau et al., 2012). Similarly, the subjects experienced extensive activation in the ventral inferior prefrontal gyrus and medial prefrontal cortex during the pain task, whereas only the inferior parietal lobule was stimulated during the exercise task (Budell et al., 2010). Therefore, it was reasonable to believe that chemotherapy further increases the difficulty for patients to decode pain stimulus signals, making the functional difference between before and after chemotherapy more obvious, which was detected in the pain empathy paradigm.

The ERP component (N1) was associated with pain perception caused by gender discrimination (Zhang et al., 2021). Participants in the pain empathy group exhibited increased discomfort and sensitivity when seeing others suffering pain, with a significant increase in the N1 amplitude (Li et al., 2020). The N1 reflected the visual component for individuals, which was closely correlated to early selective attention (Yang et al., 2017). The easier the completion of attention was, the smaller the induced amplitude was, our study found that empathic concern was reduced and the amplitude of N1 was elevated probably because more cognitive resources in emotion, attention, and visual perception need to be invested from breast cancer patients after chemotherapy. A previous study showed that P2 was associated with the feature recognition and processing of stimulation (Yuan et al., 2009; Schulz et al., 2012). In this research, we found that the amplitude of P2 was decreased for breast cancer patients after chemotherapy, and it might relate to the central nervous system lesions caused by chemotherapy drugs, making cognition and identification decline. At the same time, the main stimulus effect was obvious on P2 component, and patients were stimulated by the pain picture to produce a larger amplitude. The painful effect was arisen, which was consistent with a previous study (Ikezawa et al., 2014).

Some limitations need to be discussed in this study. First, this study was a small sample and single-center research. A larger sample size and longitudinal design should be promoted in the next study. Second, the specific brain area associated with pain empathy impairment after chemotherapy had not been approved, and further research could be combined with functional magnetic resonance imaging to remedy this deficiency.

## Conclusion

In a word, the present study provided direct evidence for pain empathy impairment in breast cancer patients after chemotherapy and preliminarily demonstrated the variation in ERP (N1, P2) components associated with pain empathy. The findings extend the scope of CRCI and furnish a theoretical basis for improving the quality of life in breast cancer patients following chemotherapy.

## Data availability statement

The original contributions presented in this study are included in the article/supplementary material, further inquiries can be directed to the corresponding author.

## Ethics statement

The studies involving human participants were reviewed and approved by the Research Ethics Committee of the Affiliated Second Hospital of Anhui Medical University, China, authorized the study (protocol 20131028). The informed consents were accomplished for all subjects. The patients/participants provided their written informed consent to participate in this study.

## Author contributions

WL performed the cognitive tests and wrote the manuscript. YL performed the data analysis and pain empathy paradigm. XD performed the data collection. GC performed the statistical analysis of the data and refined the English language. SBY and SY performed the literature review. LT performed the clinical data acquisition. HC designed the project. All authors contributed to manuscript editing.

## Footnotes

1
http://spss.en.softonic.com/

## Abbreviations

ERP: event-related potentials
IRI-C: Chinese version of Interpersonal Reactivity Index
KPS: Karnofsky Performance Status Scale
DST: digit spans tests
DSF: digit span forward
DSB: digit span backward
VFT: verbal fluency test
MMSE: mini-mental state examination.
